# Experimental Analysis of Channel Steel Member under Tension Load with Damage in the Unconnected Legs

**DOI:** 10.3390/ma16020527

**Published:** 2023-01-05

**Authors:** Ahmed M. Sayed, Hani Alanazi, Aref A. Abadel, Yousef R. Alharbi, Mohd F. Shamsudin

**Affiliations:** 1 Department of Civil and Environmental Engineering, College of Engineering, Majmaah University, Al-Majmaah 11952, Saudi Arabia; 2 Department of Civil Engineering, College of Engineering, Assiut University, Assiut 71511, Egypt; 3 Department of Civil Engineering, College of Engineering, King Saud University, P.O. Box 800, Riyadh 11421, Saudi Arabia; 4 Department of Civil Engineering, Faculty of Engineering, Universiti Malaya, Kuala Lumpur 50603, Malaysia

**Keywords:** steel damage, channel steel member, unconnected leg, damage location, tension load

## Abstract

Damage occurring to steel element structures is highly possible due to tearing ruptures, corrosion, or the adoption of sudden loads. The damage has a great effect on their capacity to bear load and the corresponding elongation, as well as the distribution of the stresses in the cross-section of the element. Therefore, in the present research, experimental tests were carried out on 15 specimens of channel steel elements with different damage ratios in the unconnected legs and at different locations along the element’s length. Through the test, the load and the corresponding elongation values were obtained for the control and damaged specimens. From the study of the different variables, it was demonstrated that the damage location does not significantly affect the load capacity, with a maximum difference of 1.9%. With the presence of the damage in only one leg at a ratio of less than or equal to 40%, the prediction of the value of the loss in the load is within the safe limit. However, if this ratio increases, there is a defect in calculating the loss in the load as it is greater than the effect of the damage. If there is any damage in the two legs of the channel together, the prediction of the loss of load is within the safe limit, where the loss is less than the effect of the damage ratio. We propose a model that can predict the capacitance of the axial load of steel channel elements through identifying the ratio of damage in the unconnected leg.

## 1. Introduction

Channel cross-section steel elements are thin-walled components that can be damaged accidentally or deliberately by tearing ruptures, the adoption of sudden loads, or corrosion. Although damaged channel steel elements are susceptible to sudden compression or tension failures that can compromise the safety of the structure, little attention has been paid to this topic to date [1]. Structural applications of the channel cross-section steel elements under tension loads are widely utilized, and although their geometries are usually relatively simple, it is very difficult to accurately predict the ultimate load capacity due to the complicated behavior exhibited by these elements [2,3]. Channel cross-section steel elements are commonly tension loaded with decentralization in perpendicular directions around the loading axis. Thus, the element is exposed to a bending moment around the loading axis [4].

While the web of the channel section is connected to the loading plates, the other legs are usually not connected. Because of the bending moment caused by decentralization, the length of these unconnected legs significantly affects the mechanical and physical properties of the element, including the maximum load capacity, final elongation, and average stress distributions [5,6,7,8]. Over the past two decades, the complex behavior of the cross-section that has unconnected legs to the loading plates has been investigated through many numerical analyses [9,10,11,12,13]. The efficiency of the steel members was studied under the influence of the presence of a bolted connection [9], the local buckling of the steel equal angle [10], the effect of uniaxial tensile loading [11], the bi-axial bending load [12], and shear tests [13]. A numerical analysis study was carried out by Sayed [14] on the single-angle cross-section, whether it was equal or unequal. Damage occurred at different ratios from 10% to 66.67% from the depth of the unconnected leg. It was concluded that if the ratio is 10%, then it does not affect the maximum load capacity of the single angle. If the damage ratio is 66.67%, then it reduces the maximum load capacity to 18%. Moreover, this damage has a significant effect on the shape of the stress distribution in the single-angle section with a discontinuous leg. Some of these studies were experimental studies that focused on the single-angle section [15,16,17,18] and the double-angles section [19]. Liu and Chantel [20] proved through an experimental analysis that the presence of eccentricity resulting from the asymmetry of the cross-section in addition to the presence of legs not connected to the loading plates affects the ultimate load, which decreased as the slenderness increased. Some studies have considered the cross-section under the influence of bending moments, including steel angle [21,22,23] and steel channels with openings in the web [24,25]. In other studies [26,27,28,29], the effect of damage occurring by opening the web for the cross-section of the steel channel was observed under the influence of the bearing loads, whether these openings were stiffened or not. The stiffened web opening can significantly improve the bearing load capacity of the channel with an edge-stiffened web opening that increased by 13.6% in comparison with that without a stiffened web opening. Furthermore, few studies have considered other construction elements, such as steel beams and steel columns, and the impact of the damage on the test results [30,31,32]. Regarding the properties of the steel element, El-Taly and Abd El-Hameed [33] studied the effect of the damage in relation to the steel beams and demonstrated that the factor of the location of the applied loads in relation to the location of the damage was the highest contributing parameter for the variation of the maximum load capacity, shear stresses, normal stresses, and vertical deformation. Furthermore, the factors of the ratio of the depth and the width of the damage were the most significant control factors affecting the tensile and compression strains and normal stresses. Some researchers have studied the damage resulting from the effect of impact load on steel elements [34,35,36]. Zeinoddini et al. [37,38] conducted a study on the dynamic behavior of axially pre-loaded tubular steel members subjected to impact damage. The extent of the damage caused by a dynamic lateral load was presumed to be largely localized, and in some cases, it can also be global. Moreover, some steel elements were studied with damage resulting from the corrosion process, and the extent of their impact on the properties and the efficiency of these elements in resisting loads were also examined [39,40,41,42,43].

**Figure 1:**
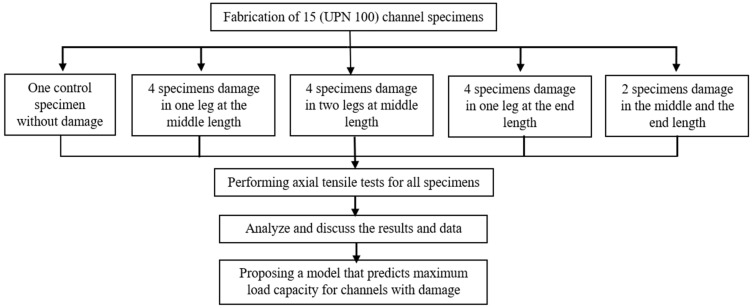
A scheme summarizing the protocol used in the current study.

Therefore, in order to elucidate the behavior of damaged channel steel elements, the current study investigated the effect of damage to the unconnected legs on the maximum load capacity, average stress distribution, and final elongation. The variables investigated were the ratio of damage depth, the location along the element’s length, and the side of the damage. These impacts are critical to improving design codes and maximizing the application range while also maintaining safety standards. Figure 1 illustrates a scheme summarizing the protocol that was used in the current study.

## 2. Experimental Tests

### 2.1. Specimen Details

**Figure 2:**
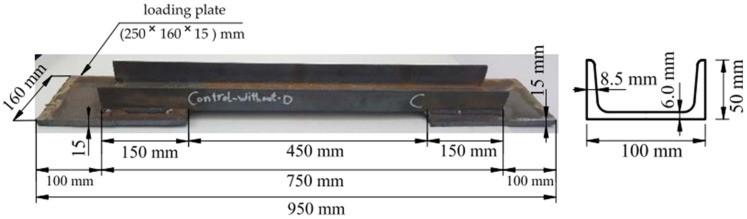
The shape and dimensions of the control specimen were used in experimental testing.

The experimental program comprised 15 specimens of steel channel of European standard (UPN 100), with a web width and thickness that was equal to 100 mm and 6.0 mm, respectively. The depth and thickness of the flanges were 50 mm and 8.5 mm, respectively, as shown in Figure 2. The total length of the specimen was 750 mm, and the effective length was 450 mm. The length of the specimens and the cross-section were chosen to match the maximum capacity of the testing machine and match what is used in steel structures. The loading plates were utilized, with the dimensions of all rectangular shapes being 250 mm × 160 mm and the thickness being 15 mm. The cross-section of the loading plate was chosen to be 2400 mm^2^ larger than the cross-sectional area of the channel in order to ensure that the failure occurred in the channel section and not in the loading plate. The loading plates were fixed to the specimens by welding. The welding consists of two lines that were equal to 150 mm (the length connected from the channel with the loading plate), with a welding size that was equal to 10 mm. In addition, there were two welding lines with a channel width that was equal to 100 mm; one was from the top at the edge of the channel with a size of 6 mm and another one was from the bottom of the channel with the edge of the loading plate with a size of 10 mm. For each specimen, two loading plates were installed at the edges of the specimen, and the fixation length was 150 mm on each side. The process of welding in the air was conducted through the use of the electric arc method (SMAW: shielded metal arc welding). The electrode types used in this process conformed to the AWS E6013 classification.

**Figure 3:**
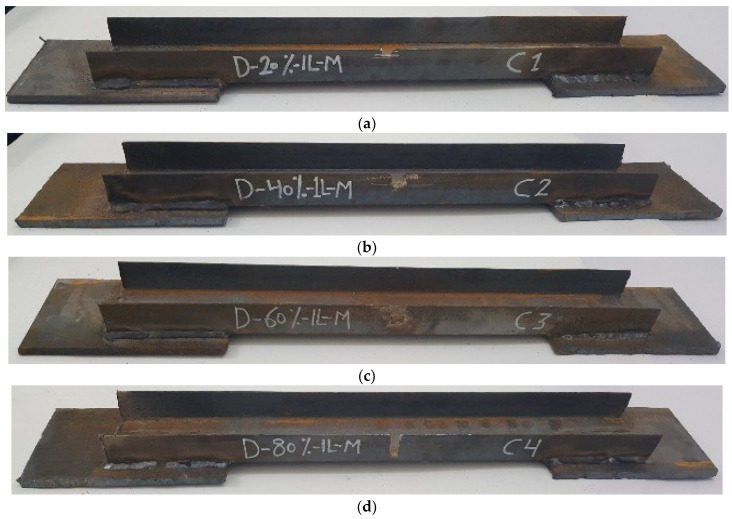
The shape of the first group with damage in one leg in the middle of the element’s length: (**a**) D-20%-1L-M; (**b**) D-40%-1L-M; (**c**) D-60%-1L-M; (**d**) D-80%-1L-M.

**Figure 4:**
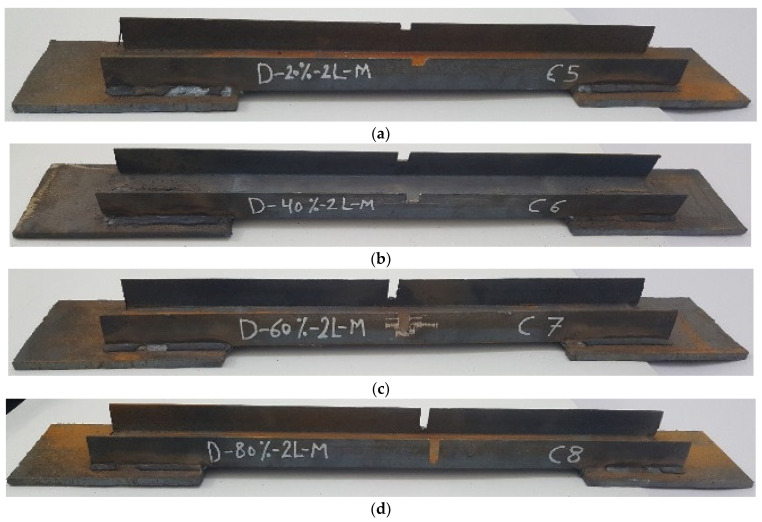
The shape of the second group with damage in two legs in the middle of the element’s length: (**a**) D-20%-2L-M; (**b**) D-40%-2L-M; (**c**) D-60%-2L-M; (**d**) D-80%-2L-M.

**Figure 5:**
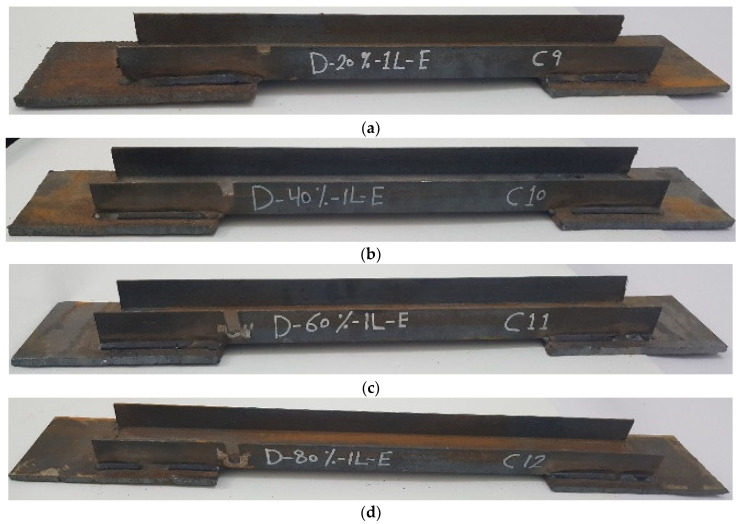
The shape of the third group with damage in one leg at the end of the element’s length: (**a**) D-20%-1L-E; (**b**) D-40%-1L-E; (**c**) D-60%-1L-E; (**d**) D-80%-1L-E.

**Figure 6:**
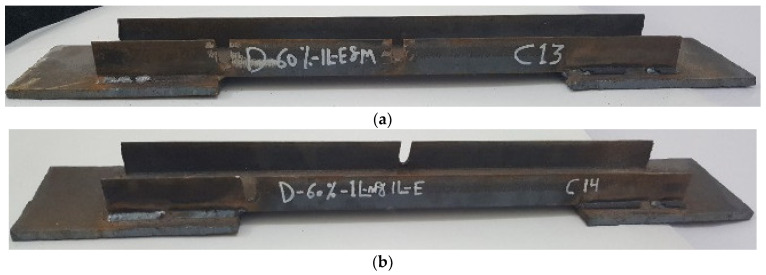
The shape of the fourth group with damage in one leg in the middle and at the end of the element’s length: (**a**) D-60%-1L-E and M; (**b**) D-60%-1L m and 1L-E.

The experimental program comprised a control specimen without damage and 14 specimens with damage in the unconnected channel legs. The specimens with damage were divided into four groups as follows: The first group had damage in one unconnected channel. The damaged area was in the middle, with damage ratios of 20%, 40%, 60%, and 80%, as shown in Figure 3. For the second group, the damage was in both the unconnected channel legs, with the same ratios of damage as in the first group, as can be seen in Figure 4. The specimens in the third group had the damage in only one unconnected channel leg at the end of the element’s length, as shown in Figure 5. The specimens of the fourth group had 60% damage in one leg only at the cross-section. However, there were two damaged sections, one was in the middle and the other one was at the end of the element’s length. Furthermore, one of the specimens had damage in the same unconnected leg on one side, and the other specimen had damage on two different sides, as shown in Figure 6. This damage had a rectangular shape with a depth that was equal to the required damage ratio and a fixed width that was equal to 10 mm, which was achieved through the use of an electric cutter. Three conditions must be taken into account when one is producing damage. Firstly, the damage should originate on the free end of the unconnected channel leg. Secondly, the direction of the damage should be perpendicular to the direction of the length of the element. Thirdly, if there is damage in more than one location, they must have the same ratio. The specimens were named, and some names were shortened to symbols, for example, D-20%-1L-M, D-20%-2L-M, and D-20%-1L-E, where, D = damage, 20% = the damage rate is 20% of the depth of the unconnected channel leg, 1L = damage to one leg of the channel, M = damage to the middle of the element’s length, 2L = damage to two legs of the channel, and E = damage to the end of the element’s length.

### 2.2. Instrumentation and Test Setup

**Figure 7:**
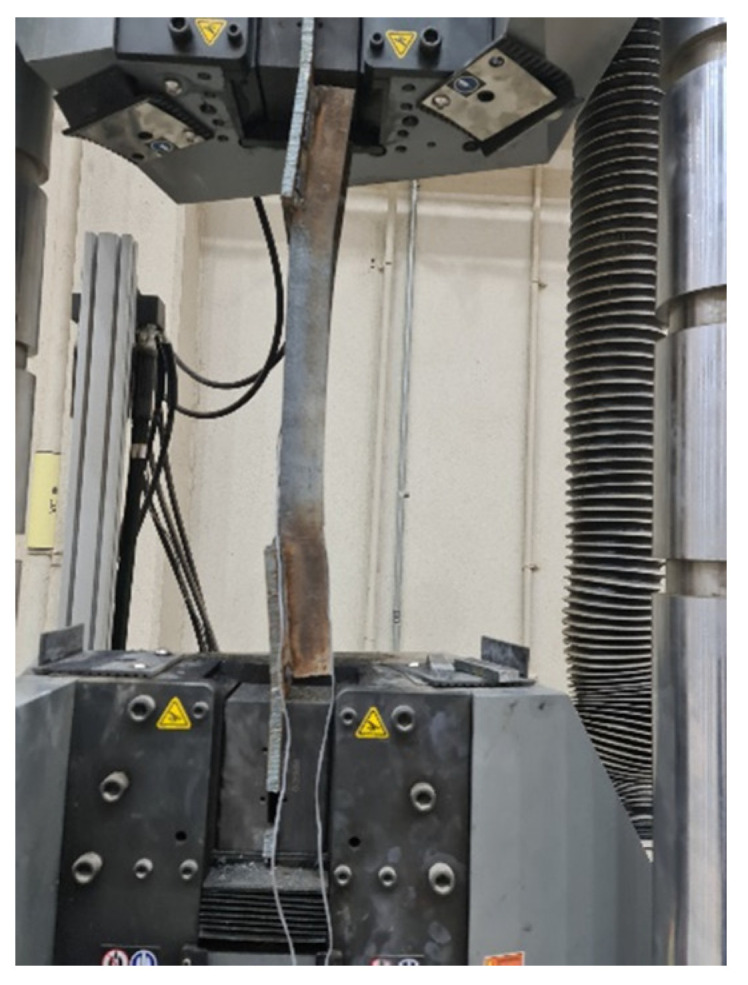
The method of attaching the sample to the tension machine employed in the tests.

Fifteen steel channel UPN specimens were tested using a tensile loading machine (Civil Engineering Department Lab, King Saud University, Riyadh, Saudi Arabia) with a capacity of 1000 kN, as presented in Figure 7. The testing machine had a fixed upper jaw and a mobile lower jaw. Tensile tests were conducted for each specimen at a tensile load rate of 5.0 kN/min for all of the specimens. A computer-aided data acquisition system was employed for monitoring the loads and displacements.

### 2.3. Determining the Properties of the Steel Used

All of the specimens were manufactured from a single member with a length of 12 m in order to ensure that all of the steel specimens had the same properties so that the comparison would be correct and accurate. Three tensile specimens were taken from the web of the channel cross-section, with a length of 200 mm and net thickness of 6.0 mm. The standard dimensions follow the ASTM A370 standard [44,45]. The tensile test was carried out on the specimens, and the load and the corresponding elongation values were measured. The strain was measured through the use of a strain gauge, which was placed along the middle of the specimen’s length. The values of yield stress, ultimate stress, strain at failure, and modulus of elasticity were calculated. The average values for the three specimens are included in Table 1.

## 3. Experimental Results and Discussion

The different variables, including the presence of damage in one leg of the steel channel cross-section or the presence of damage in both legs together at one cross-section and the use of different damage ratios (from 20% to 80%) from the depth of the unconnected legs, as well as the presence of damage in a different location along the element’s length, were studied under the influence of the axial tensile load. The loads and corresponding elongations were recorded through the use of the test machine. The impact of these variables on the maximum load capacity, the value of elongation, and the distribution of stresses on the cross-section of the steel element were analyzed.

### 3.1. Damage Impact on Load–Elongation Relationships

**Figure 8:**
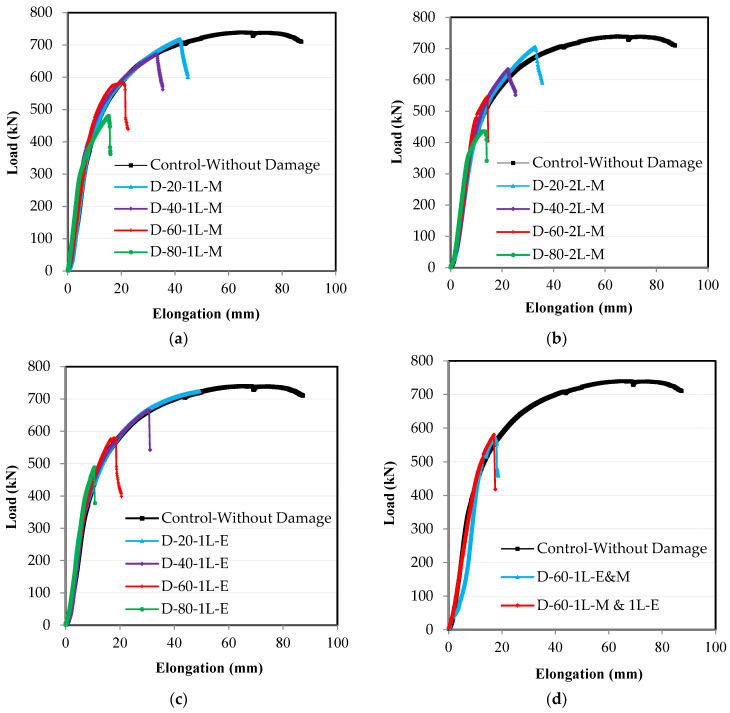
Experimental test results of load-elongation relationships: (**a**) damage in the middle of the length in one leg; (**b**) damage in the middle of the length in two legs; (**c**) damage at the end of the length in one leg; (**d**) damage at the end and middle of the length in one leg.

A major relationship determining the efficiency and resistance of the steel elements subjected to an axial tensile load is the load–elongation relationship. The axial tensile test was carried out on all of the specimens under the same conditions as for the ends. The load and corresponding elongation were measured using the testing machine. The relationship between the load and the corresponding elongation was drawn for all of the specimens, as shown in Figure 8. It was demonstrated that the load–elongation relationship of the control specimen continues to increase until the maximum load was applied, and then, the relationship began to decrease until it reached the failure point (Figure 8). In the rest of the damaged specimens, the relationship continued to increase until the maximum load was applied, then, suddenly, the failure occurs. It was mostly observed that all of the specimens had the same curved shape as the control specimen until they reached the failure point, but as the damage ratio increased in the unconnected steel channel leg, the maximum load, and the corresponding elongation decreased. From the observation that the shape of the failure changed from the ductility failure of the control specimen to the sudden failure of the damaged specimens, there is a real danger in the behavior of the steel elements that have incurred damage to the unconnected leg. Therefore, the maximum design load that can be carried out by this steel element must be considered so that this type of failure does not occur. From these relationships, which are shown in Figure 8, the effect of the damage ratio in the unconnected leg can be observed on the ultimate load and the corresponding elongation, as well as the ratios between these values for specimens with damage in relation to the control specimen, as presented in Table 2.

### 3.2. Damage Impact on Cross-Section Stresses

**Figure 9:**
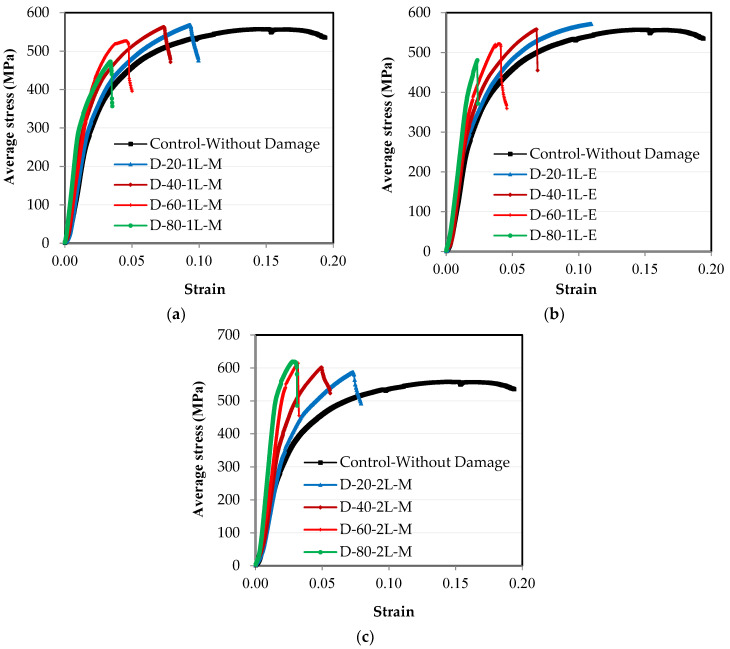
Experimental average stress–strain engineering relationships: (**a**) damage in the middle of the length in one leg; (**b**) damage at the end of the length in one leg; (**c**) damage in the middle of the length in two legs.

From the relationship between the load and elongation, which were measured using the test machine, the engineering stress–strain relationships were obtained by dividing the load on the cross-sectional area to obtain the stress and dividing the elongation on the length of the element to obtain the strain, as shown in Figure 9. Over the length of the steel element, there are two stress values according to the location of the cross-section, where there is stress on the original section without damage, and there is another stress on the damaged section, where the area of the section decreases due to the damage. The relationship between the engineering stress and strain over the entire element’s length except for the damaged section is the same as that between the load and elongation, as shown in Figure 8. Meanwhile, the engineering stress–strain relationship at the damaged section varies according to the ratio of damage, as presented in Figure 9. For all of the specimens that were subjected to damage, as the damage ratio increases in the unconnected channel leg, the strain on the entire length of the steel channel element decreases, whether the damage is in one leg or both legs or in the middle of the element’s length or at the end (Figure 9).

**Figure 10:**
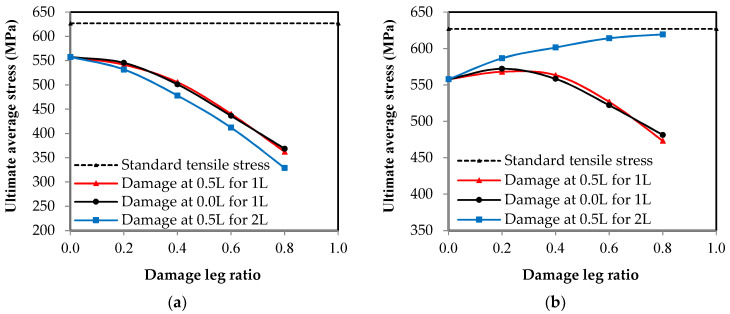
The relationship between the average ultimate stress and the damage ratio in the unconnected leg. (**a**) Stresses on the original section; (**b**) stresses on the damage section.

**Figure 11:**
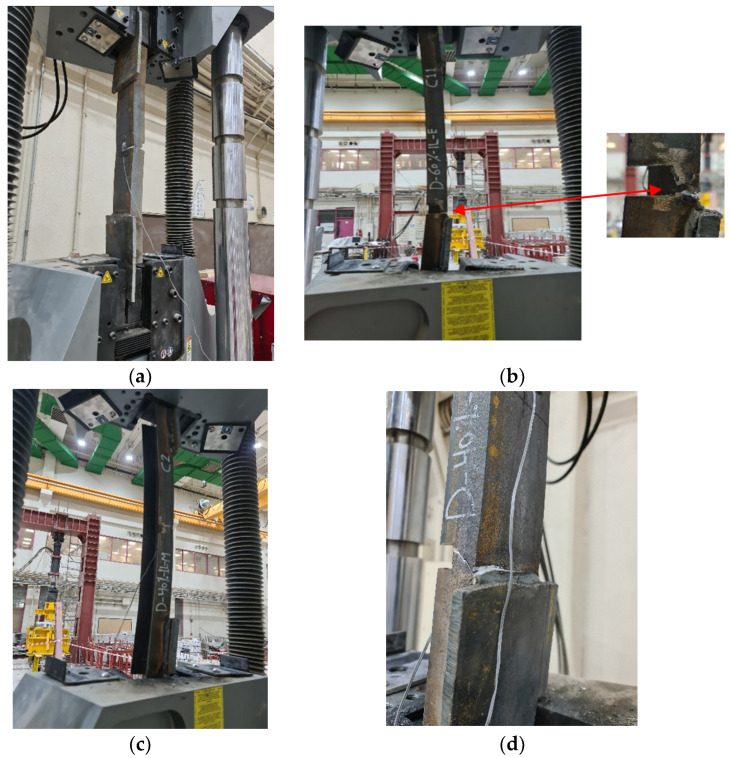
The pattern of failure shape and location for the channel damage in the unconnected leg: (**a**) D-20%-1L-M; (**b**) D-60%-1L-E; (**c**) D-40%-1L-M; (**d**) D-40%-1L-E.

**Figure 12:**
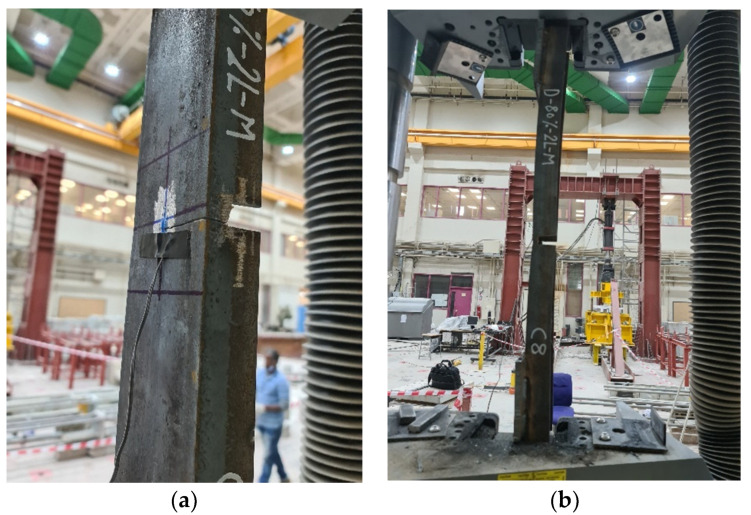
The pattern of failure shape and location for the channel damage in the two unconnected legs: (**a**) D-60%-2L-M; (**b**) D-80%-2L-M.

As for stress, the results differ according to the ratio of damage in one leg or in both legs of the channel section. In order to clearly understand this effect, the relationship between the maximum average stress and the ratio of damage for all of the specimens is represented in Figure 10. In Figure 10a, the value of the stresses on the cross-section over the entire length of the steel channel element, except for the section that was exposed to damage, decreases as the damage ratio increases in the unconnected leg, whether the damage is in one leg or both legs or in the middle of the length or at the end. However, the value of this reduction has a non-linear relationship, where the value of the reduction is small when the percentage of the damage has a value of up to 40%, and it increases as the damage ratio increases. In Figure 10b, the value of the stresses on the cross-section exposed to damage varies according to the location of the damage in one leg or in both legs. It was found that if the damage is in one leg only, whether it is in the middle of the length or at the end, the maximum average stress decreases as the percentage of damage increases. If the damage is in both of the unconnected channel legs, the maximum average stress increases with the increase in the damage ratio that was incurred in the cross-section. The reason for this result is that when the damage is found in only one leg, the stress concentrates at the corner where the damaged leg connects with the connected channel web. Therefore, the failure occurs at this point of the cross-section, as shown in Figure 11. Meanwhile, the other leg, which has not been exposed to damage, has a distribution of non-uniform stresses, as presented in some studies [2,6,14]. This is due to the eccentricity of the load being in the longitudinal direction in addition to the eccentricity of the load being in the transverse direction as a result of the damage ratio being on one side only. Therefore, the average stress on the cross-section decreases as the damage ratio increases in one leg of the cross-section. When there damage has occurred to the two unconnected channel legs, the stresses are redistributed along the cross-section. The greater the damage ratio is, then the lower the value of decentralization for the section is, which means that there is a semi-regular stress distribution. Moreover, in order for the tensile failure to occur, the stresses must reach the maximum for the steel material used. Therefore, as the damage ratio increases in both of the unconnected legs, the average actual stress at the damaged section increases (i.e., the damage ratio in both of the legs improves the shape of the distribution of stresses in the cross-section). As a result, the web tensile failure occurs without the stress concentrating in a particular corner of the cross-section, as shown in Figure 12. Generally, in all of the specimens, whether it is the control or the damaged specimens, the ultimate average stress in the cross-section does not reach the ultimate stress of the steel used, which is 625.89 MPa. When the percentage of damage is 80% of the depth of one unconnected leg, its maximum average stresses reach 473.30 MPa, while the stresses in the damage in the two legs reach 619.43 MPa. Furthermore, the maximum average stress over the entire length of the element, except for the damaged section, decreases significantly until it may reach a stress value that is less than the stress of yielding to the steel being used, which is 398.61 MPa. This is the reason why the elongation is greatly reduced in the damaged specimens.

### 3.3. Damage Impact on Member Axial Load Capacity

**Figure 13:**
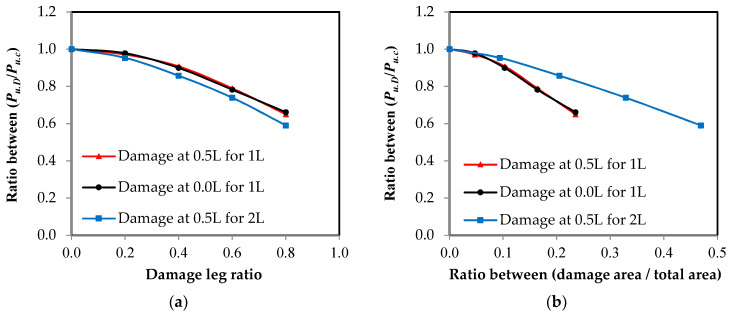
The relationship between the ultimate load ratio and the damage ratio in the unconnected leg. (**a**) Damage ratio from leg depth; (**b**) damage ratio from the cross-sectional area.

**Figure 14:**
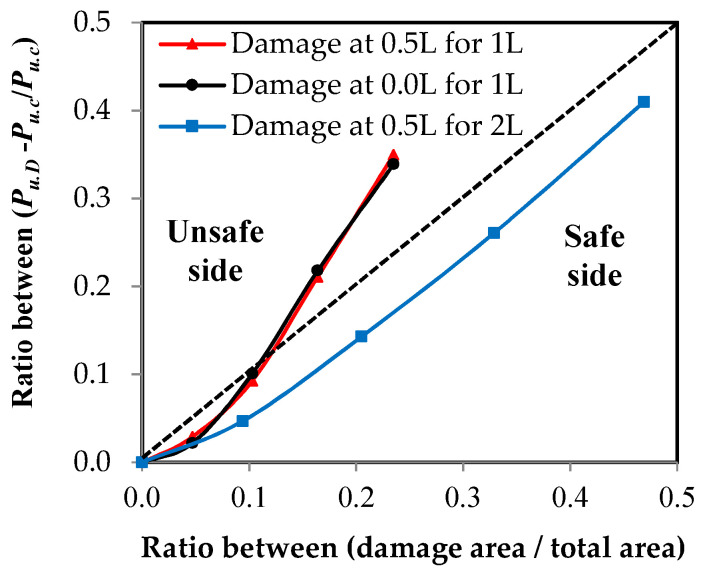
The relationship between the ultimate load reduction ratio and the damage ratio from the cross-sectional area.

Using the relationship between the load and the elongation, which were measured using the testing machine for all of the specimens, the relationship between the load ratio of the damaged elements to the control element and the damage ratio in the unconnected leg is represented in Figure 13. These relationships were affected by two factors: the damage ratio in the unconnected leg related to the total depth and the location of the damage in relation to the length of the element. The damage ratio is represented in two ways as follows: Figure 13a shows the representation of damage as a ratio of the depth of the unconnected leg and Figure 13b represents the damage as a ratio of the cross-sectional area of the element. In terms of the type of channel UPN, it was found that the thickness of the unconnected leg was not fixed over the entire depth, so the reduction of the area of the cross-section was irregular according to the ratio of damage in the depth. As shown in Figure 13, as the value of the damage increases, the maximum capacity of the axial load of the steel channel element decreases. When the damage ratio in one unconnected leg is 80%, the reduction of the carrying capacity reaches 65% and 66% of the total load of the non-damaged element when the damage is in the middle or at the end of the steel element’s length, respectively. However, when the damage in the two unconnected channel legs is 80%, the reduction reaches 59%. From the shape of the curves, it was found that the value of reduction at the beginning of the damage is small, and as the damage ratio increases, the percentage of reduction becomes large. The relationships between the effect of the reduction of the damaged cross-sectional area and the percentage of the reduction of the maximum load capacity are presented in Figure 14. A 45 degree inclined line has been drawn, which is a line separating the safe or unsafe sides with a reduced ratio in the maximum axial load capacity for the damaged steel channel sections (Figure 14). It was observed that when there is damage in the two unconnected legs, the reduction of the load ratio is smaller than that in the cross-sectional area, so all of the cases are on the safe side of the design. When the percentage of damage in the two unconnected legs reaches 80% of the depth, which is equivalent to 46.9% of the cross-sectional area, the reduction of the percentage of load reaches 41%. When the damage happens to one leg only, whether this damage is in the middle or at the end of the element’s length or the damage ratio is up to or equal to 40% of the depth of the unconnected channel leg, which is equivalent to a damage rate of 10.30% of the cross-sectional area, the reduction of the load ratio is less than that in the cross-sectional area, which is, on the safe side of the design. However, when the damage ratio exceeds these values, the reduction ratio in the load begins to increase and it moves to the unsafe side of the design. When the percentage of damage reaches 80% of the depth of the unconnected leg, which is equivalent to 23.5% of the cross-sectional area, the reduction of the percentage of load for the damaged elements in the middle or at the end of its length reaches 35% and 33.9%, respectively. This is due to the presence of stress concentration at the corner of contact of the damaged leg with the web of the steel channel section, which accelerates the failure of the element at a load of less than the equivalent load for the actual cross-sectional area. Therefore, this deficiency must be considered when one is designing steel elements that may contain damage to one unconnected leg in that are ratios greater than 40%.

### 3.4. Damage Impact on Member Elongation

**Figure 15:**
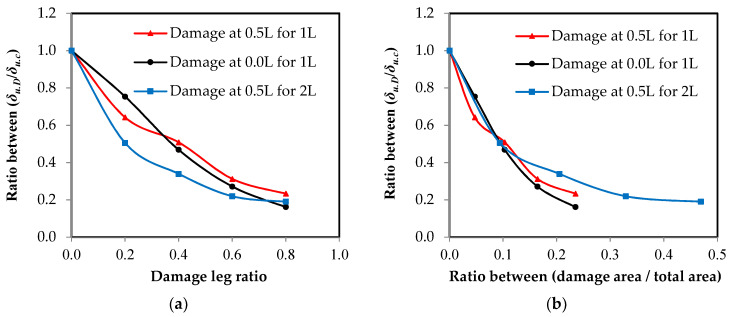
The relationship between the final elongation ratio and the damage ratio in the unconnected leg. (**a**) Damage ratio from leg depth; (**b**) damage ratio from the cross-sectional area.

The relationship between the elongation ratio of the damaged elements to the control element and the damage ratio in the unconnected leg is represented in Figure 15. It is clear that as the damage ratio increases, the percentage of total elongation of the element decreases. Moreover, it was found that when a damage percentage is equal to 80% of the total depth of the unconnected leg, the average elongation percentage in the reduction reaches 19.33% of the elongation of the undamaged element. It was also obvious that when there is any ratio of damage, it directly affects the total elongation of the element, even if this damage is small. The reason for this reduction of the total elongation of the element when there is damage is that the stresses on the cross-section over the entire length of the element, except for the damaged section, do not reach the maximum stress for the steel used and that most of the elongation occurs at the damaged section of the element. This result is consistent with that of [14]. Using the relationships in Figure 15a, models have been proposed through the use of a polynomial equation that can predict the total elongation values of the damaged element by identifying the damage ratio in the unconnected leg.

For steel channel UPN elements with damage in one leg in the middle of the channel length with a correlation coefficient of *r* = 0.994:

(1) $$\delta_{u.d}=\delta_{u.c}\left(0.8857\times D_{r}{}^{2}-1.6406\times D_{r}+1.0\right).$$

For steel channel UPN elements with damage in one leg at the end of the channel length with a correlation coefficient of *r* = 0.998:

(2) $$\delta_{u.d}=\delta_{u.c}\left(0.6464\times D_{r}{}^{2}-1.5971\times D_{r}+1.0\right).$$

For steel channel UPN elements with damage in two legs in the middle of the channel length with a correlation coefficient of *r* = 0.992:

(3) $$\delta_{u.d}=\delta_{u.c}\left(1.7464\times D_{r}{}^{2}-2.3501\times D_{r}+1.0\right).$$

In the above equations, *δ_u_*_.*d*_ is the final elongation in the steel channel element after damage, *δ_u.c_* is the final elongation in the steel channel element without damage, and *D_r_* is the damage leg ratio.

### 3.5. Impact of Damage Location in Relation to Element’s Length

**Figure 16:**
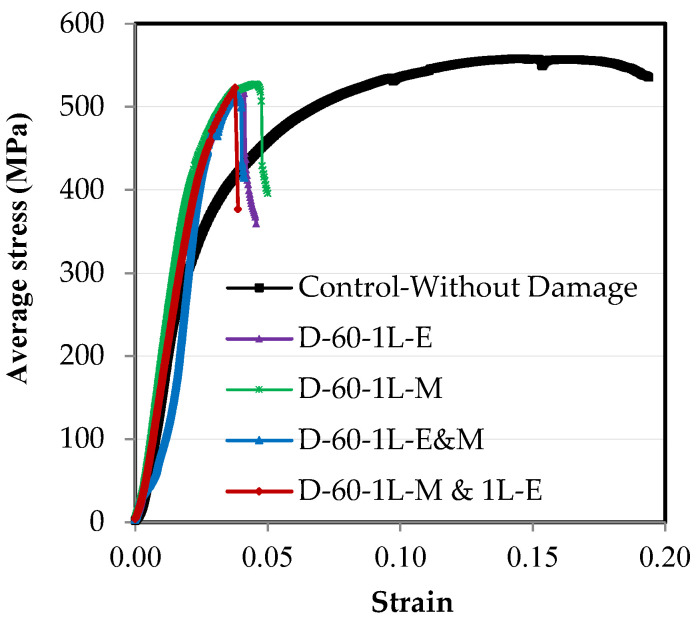
The effect of the damage location on the stress–strain relationships of the steel element.

**Figure 17:**
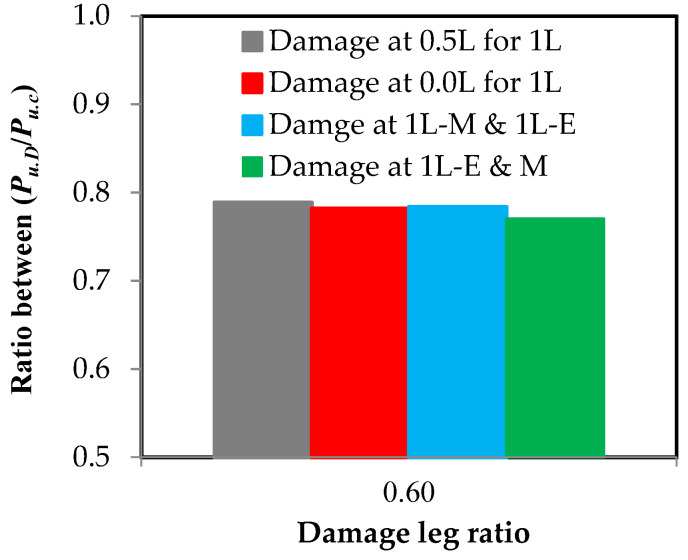
The effect of the damage location on the reduction ratio in the load capacity of the steel element.

From Figure 16 and Figure 17, it is clear that the damage location in relation to the length has a weak effect on the properties of the steel element. In Figure 16, it was found that when there is damage at 60% of the depth of the steel channel element that is not connected, the relationship between the stress and the strain is very close despite the damage being in the middle or at the end of the element’s length or in the middle and at the end together. Additionally, in Figure 17 which shows the effect of the damage location on the maximum load capacity of the steel channel element, it was found that the maximum difference between the load reduction ratios where the damage is in the middle or at the end or both together on one or two different sides is 1.9%. This result is fully consistent with the study conducted by Sayed [14] who examined the damage in the single unconnected angle leg at three locations along 0.0, 25%, and 50% of the element’s length, with a maximum difference being between the percentages of load loss of 2.0%. This completely agrees with the result in the current research.

## 4. Predicting the Load Capacity of Steel Channel Element with Damage in the Unconnected Leg

Figure 13 shows the relationship between the reduction of the load ratio as a result of the presence of damage in the unconnected leg and the damage ratio causing this. Through the use of polynomial equations, it was possible to obtain models that can predict the value of the maximum axial load capacity of the damaged steel channel UPN element by identifying the value of damage, whether it was a ratio of the unconnected leg depth or a ratio of the cross-sectional area, as well as the maximum load capacity of the steel element without damage. The models were divided based on the location of the damage in relation to the element’s length, as well as whether the damage was in one leg or both legs together.

From Figure 13a, when the damage is used as a ratio of the unconnected leg depth:

For steel channel UPN elements with damage in one leg at the end of the channel length with a correlation coefficient of *r* = 0.998:

(4) $$P_{u.d}=P_{u.c}\left(-0.4214\times D_{r}{}^{2}-0.0999\times D_{r}+1.0\right)$$

For steel channel UPN elements with damage in one leg in the middle of the channel length with *r* = 0.996:

(5) $$P_{u.d}=P_{u.c}\left(-0.4893\times D_{r}{}^{2}-0.0496\times D_{r}+1.0\right)$$

For steel channel UPN elements with damage in two legs in the middle of the channel length with *r* = 0.999:

(6) $$P_{u.d}=P_{u.c}\left(-0.4036\times D_{r}{}^{2}-0.1941\times D_{r}+1.0\right)$$

From Figure 13b, when the damage is used as a ratio of the cross-sectional area:

For steel channel UPN elements with damage in one leg at the end of the channel length with *r* = 0.997:

(7) $$P_{u.d}=P_{u.c}\left(-3.0306\times D_{r}{}^{2}-0.7922\times D_{r}+1.0\right)$$

For steel channel UPN elements with damage in one leg in the middle of the channel length with *r* = 0.999:

(8) $$P_{u.d}=P_{u.c}\left(-3.9242\times D_{r}{}^{2}-0.5995\times D_{r}+1.0\right)$$

For steel channel UPN elements with damage in two legs in the middle of the channel length with *r* = 0.999:

(9) $$P_{u.d}=P_{u.c}\left(-0.6739\times D_{r}{}^{2}-0.572\times D_{r}+1.0\right)$$

where *P_u_*_.*d*_ is the remaining axial load in the damage steel channel element, *P_u_*_.*c*_ is the load in the steel channel element without damage, and *D_r_* is the damage ratio. All of these relationships exhibited a very high correlation coefficient of *r* = 0.99, indicating an accurate prediction of the damage impact on the strength of the steel element.

**Figure 18:**
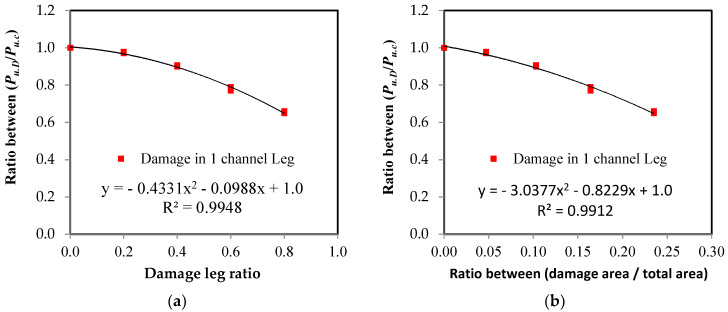
The average relationship between ultimate load ratio and damage ratio in the unconnected leg. (**a**) Damage ratio from leg depth; (**b**) damage ratio from the cross-sectional area.

The experimental test results demonstrated that the effect of the damage location on the load capacity was not as significant, with a maximum difference of 1.9%. Therefore, in order to facilitate the prediction of the value of residual axial load in the steel channel element with damage in the unconnected leg, a single relationship is represented in Figure 18, including all of the samples in which damage occurred in one leg, whether it was in the middle or at the end of the element’s length, or both. The average of the two equations was taken and combined into one equation.

From Figure 18a, when the damage is used as a ratio of the unconnected leg depth:

For steel channel UPN elements with damage in one leg with a correlation coefficient of *r* = 0.997:

(10) $$P_{u.d}=P_{u.c}\left(-0.4331\times D_{r}{}^{2}-0.0988\times D_{r}+1.0\right)$$

From Figure 18b, when the damage is used as a ratio of the cross-sectional area with *r* = 0.996:

(11) $$P_{u.d}=P_{u.c}\left(-3.0377\times D_{r}{}^{2}-0.8229\times D_{r}+1.0\right)$$

These proposed models are used only for the elements with the UPN channel cross-section, whereas during the study by Sayed [14], models were proposed which can predict the maximum capacity with damage, but they are used only for single-angle cross-sections. Therefore, a model must be available for each different cross-section, as is evident in this study and the previous study [14].

## 5. Conclusions

Damage can occur in a steel section as a result of many factors, including natural and executive ones. The damage can affect the efficiency of the steel element in carrying external loads based on the percentage of damage and the type of load. Therefore, experimental tests were carried out in order to predict the maximum axial load capacity of UPN channel steel members with damage to the unconnected legs. The test was conducted on 14 specimens with damage to the unconnected legs with different ratios and in different locations of the element’s length in addition to a control specimen without damage. The following conclusions can be drawn from the obtained results and the analysis:

- The impact of the damage in the two unconnected legs at any ratio, or the damage at a ratio of less than or equal to 40% in only one leg, causes a reduction of the load capacity that is less than that of the reduced area of the cross-section (i.e., a safe design).
- A damage effect that is greater than 40% on one leg only causes a reduction of the load capacity that is greater than that of the reduced area of the cross-section (i.e., unsafe design).
- Damage in only one leg causes an additional decentralization of the cross-section in the transverse direction, resulting in a concentration of stresses at the corner of the connection between the damaged leg and the web of the channel section, which accelerates the failure with a carrying capacity that is less than the design load of the cross-section.
- As the damage ratio increases in the two legs together, the eccentricity of the cross-section reduces, improving the shape of the stress distributions through the cross-section.
- The damage location along the element’s length does not significantly impact the measured axial load capacity, with the maximum difference being 1.9%.

These results can be used to predict the maximum capacity of the channel section when there is damage to the unconnected legs or when these elements are reused again with the presence of this damage.

## Figures and Tables

**Table 1:** Summary of the experimental tensile test results for the steel plate.

Steel Plate Specimens	Net Width (mm)	Yielding Stress *σ_y_* (MPa)	Ultimate Stress *σ_u_* (MPa)	Strain at Failure *ε_f_*	Modulus of Elasticity *E* (GPa)
S-1	40.1	389.27	626.94	0.18752	204.74
S-2	40.3	399.84	619.22	0.20113	209.23
S-3	39.8	406.72	631.51	0.21001	200.19
Average	---	398.61	625.89	0.19955	204.72

**Table 2:** Experimental test results of steel channel specimens with different damage ratios.

Channel Specimen	Damage at Unconnected Leg (%)	Damage Location from Length	Ultimate Load *P* (kN)	*P_U_*_.*D*_/*P_U.C_*	Elongation at Ultimate *δ* (mm)	*δ*_F.D_/*δ*_F.C_	Average Stresses at the Damage Cross-Section		
							Area (mm^2^)	*A*_D_/*A*_C_	Stress (MPa)
Control-without-D	without	—	739.61	1.000	65.35	1.000	1326	1.000	557.78
D-20%-1L-M	20	0.50L	718.17	0.971	41.98	0.642	1264	0.953	568.17
D-40%-1L-M	40	0.50L	670.56	0.907	33.23	0.508	1190	0.897	563.50
D-60%-1L-M	60	0.50L	583.68	0.789	20.36	0.312	1108	0.836	526.79
D-80%-1L-M	80	0.50L	480.40	0.650	15.21	0.233	1015	0.765	473.30
D-20%-2L-M	20	0.50L	705.20	0.953	32.97	0.505	1202	0.906	586.69
D-40%-2L-M	40	0.50L	634.00	0.857	22.18	0.339	1054	0.795	601.52
D-60%-2L-M	60	0.50L	546.65	0.739	14.32	0.219	890	0.671	614.21
D-80%-2L-M	80	0.50L	436.08	0.590	12.42	0.190	704	0.531	619.43
D-20%-1L-E	20	L	723.39	0.978	49.22	0.753	1264	0.953	572.30
D-40%-1L-E	40	L	664.54	0.899	30.61	0.468	1190	0.897	558.44
D-60%-1L-E	60	L	578.51	0.782	17.74	0.271	1108	0.836	522.12
D-80%-1L-E	80	L	488.64	0.661	10.50	0.161	1015	0.765	481.42
D-60%-1L-E and M	60	0.5L + L	569.36	0.770	17.43	0.267	1108	0.836	513.86
D-60%-1L m and 1L-E	60	0.5L + L	579.80	0.784	16.94	0.259	1108	0.836	523.29

## Data Availability

The data presented in this study are available on request from the corresponding author.
